# Application of Human Epineural Patch (hEP) as a Novel Strategy for Nerve Protection and Enhancement of Regeneration After Nerve Crush Injury

**DOI:** 10.3390/biomedicines13071633

**Published:** 2025-07-03

**Authors:** Katarzyna Kozlowska, Weronika Radecka, Sonia Brodowska, Lucile Chambily, Dominika Kuc, Amber Lopez, Maria Siemionow

**Affiliations:** Department of Orthopaedics, University of Illinois at Chicago, Chicago, IL 60607, USA; kozlowska-k@wp.pl (K.K.); wradec3@uic.edu (W.R.); soniaa.bro@gmail.com (S.B.); lucile.chambily@gmail.com (L.C.); d.kuc06@gmail.com (D.K.); ambernl2@uic.edu (A.L.)

**Keywords:** human epineural patch (hEP), human amniotic membrane (hAM), nerve crush injury, peripheral nerve regeneration, nerve protection, functional outcomes, regenerative medicine

## Abstract

**Background**: Numerous experimental studies aim to improve outcomes of peripheral nerve repair following trauma. This study evaluates the efficacy of the human epineural patch (hEP) compared to the human amniotic membrane (hAM) in promoting nerve regeneration following sciatic nerve crush injury. **Methods**: Thirty-six athymic nude rats were divided into three groups (*n* = 12 per group) following nerve crush: (1) an unprotected injury site; (2) crush injury wrapped with hEP; and (3) crush injury wrapped with hAM. Animals were assessed over 6 or 12 weeks post-injury. Evaluations included motor recovery (Toe-Spread test), sensory recovery (Pinprick test), muscle denervation atrophy (the gastrocnemius muscle index (GMI)), histomorphometry (myelin thickness, axonal density, fiber diameter, and percentage of myelinated fibers), and immunofluorescence (GFAP, Laminin B, NGF, S-100, VEGF, vWF, HLA-DR, and HLA-I) assessments. **Results**: The hEP group showed superior motor recovery, axonal density and higher GMI values compared to the hAM and control groups. The increased expression of neurogenic and angiogenic markers highlighted its neuroregenerative potential. Negligible HLA-DR and HLA-I expression confirmed the lack of hEP and hAM immunogenicity. **Conclusions**: The application of hEP following sciatic nerve crush injury facilitated nerve regeneration, improved functional outcomes, and offered a viable alternative to hAM. Structural stability and the regenerative capacity position hEP as a new, promising off-the-shelf product for nerve regeneration.

## 1. Introduction

Peripheral nerve and soft tissue injuries significantly contribute to the morbidity and disability of patients due to traffic accidents, war conflicts, natural disasters, and iatrogenic factors [1,2]. Traumatic nerve injuries profoundly impact patients’ daily activities and quality of life, limiting their ability to fulfill professional duties and recreational activities. Despite early diagnosis, nerve recovery is prolonged and functional outcomes are frequently unsatisfying [3]. According to recent studies, fewer than half of individuals with traumatic peripheral nerve injuries undergo surgical intervention, and only 40–50% experience successful functional recovery [4]. These injuries carry substantial clinical significance due to their impact on both sensory and motor function, often resulting in impaired mobility, a loss of independence, and chronic pain if inadequately managed. This creates a significant burden on both patients and healthcare systems.

Although microsurgical techniques have advanced, treatment options remain limited and often fail to provide satisfactory outcomes in clinical practice. Recovery is often incomplete—particularly in complex cases or when surgical repair is delayed—and is frequently complicated by scar formation, local fibrosis, and inflammation [5,6,7]. Moreover, nerve regeneration is typically slow and unpredictable, resulting in prolonged rehabilitation and inconsistent results. Emerging innovations, including bioengineered scaffolds or the targeted delivery of neurotrophic factors, offer new avenues in the field of nerve repair. Materials used for nerve repair and protection, including silicone sheets, collagen, fat grafts, vascularized fat, muscle flaps, vein grafts, and omentum, present various advantages and disadvantages, indicating the need for further research to improve nerve regeneration under unfavorable healing conditions [8].

One of the commercially available materials used for nerve protection after injury is the human amniotic membrane (hAM), which supports nerve regeneration processes due to its antifibrotic and anti-inflammatory properties, as well as low immunogenicity [8,9,10]. Recent studies demonstrate that hAM reduces perineural adhesions, enhances wound healing, and restricts scar tissue formation. However, the specific mechanism of hAM action in axonal regeneration is still poorly understood [11,12,13]. Moreover, there are still several challenges for the routine hAM application, including demanding preservation and storage processes, inherent heterogeneity, and restricted mechanical strength [14].

In response to the limitations of current nerve repair techniques, we have developed the human epineural patch (hEP) derived from the human epineurium as a novel approach for peripheral nerve regeneration after trauma. The hEP product was developed following extensive research testing epineurium-based constructs, including human epineural jackets, conduits (hEC), and patches, which have demonstrated significant neuroregenerative potential in the preclinical studies [5,15,16,17,18,19,20,21,22,23].

In this study, we introduce a novel strategy for enhancing nerve regeneration after trauma by applying hEP to the nerve crush injury site. The efficacy of hEP is compared with commercially available hAM, hypothesizing that hEP will create a more conducive microenvironment for nerve repair, minimize adhesions and scar tissue formation, and foster more effective nerve regeneration, ultimately leading to improved functional recovery.

## 2. Materials and Methods

### 2.1. Experimental Animals

Animal care and experimental protocols were approved by the Institutional Animal Care and Use Committee (IACUC) of the University of Illinois at Chicago, accredited by the American Association for the Accreditation of Laboratory Animal Care (AAALAC). In this experimental study, a total of 36 eight-week-old male athymic homozygous nude rats (*Crl:NIH-Foxn1^rn^^u^*, Charles River Laboratories, Wilmington, MA, USA), weighing 150–250 g were used. Rats were maintained in room temperature-controlled cages, on a 14/10 light–dark cycle, with access to water and rodent chow ad libitum. All animals received humane care in compliance with the ‘Principles of Laboratory Animal Care’ formulated by the National Society for Medical Research and the ‘Guide for the Care and Use of Laboratory Animal Resources’ published by the US National Institutes of Health.

### 2.2. Human Epineural Patch Creation

**Figure 1:**
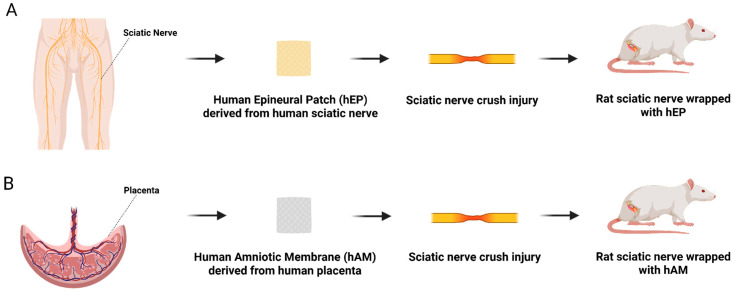
Schematic representation of the study design evaluating nerve regeneration following crush injury in the athymic nude rat model. The diagram illustrates the creation and application of hEP as an innovative therapeutic approach for promoting nerve regeneration after crush injury.

**Figure 2:**
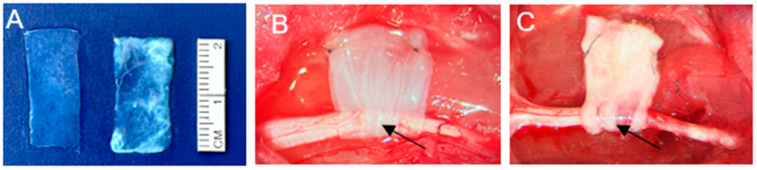
Application of hAM and the created hEP at the sciatic nerve crush site. (**A**) Dimensional assessment of the patches, each measuring 1 cm × 2 cm in length: (left) hAM and (right) hEP. (**B**) Sciatic nerve crush injury wrapped with hAM. (**C**) Sciatic nerve crush injury wrapped with hEP. Black arrows indicate the location of the injury at the crush site.

For this experimental study, human sciatic nerves were obtained from the Musculoskeletal Transplant Foundation (MTF). Upon delivery, the nerves were maintained in a sterile environment on dry ice and immediately transferred into a −86 °C Glacier^®^ Ultralow Temperature Freezer (Thermo Fisher Scientific, Waltham, MA, USA). Preparation of the human epineural patch (hEP) (Figure 1A) began with controlled thawing using a 38 °C circulating water heating system (T/Pump^®^, Gaymar Industries, Orchard Park, NY, USA). To ensure reproducibility and potential translational application, several critical parameters in the use of human epineural patch (hEP) were standardized. Viable hEP was trimmed to uniform dimensions of 2 cm × 1 cm to ensure consistent coverage of the nerve injury site and stored in saline until implantation at the injury site in the rat sciatic nerve model (Figure 2A). Following previously optimized protocols, the nerves were processed under 20× magnification using a surgical microscope (Wild M-691, Leica Microsystems, Wetzlar, Germany). The epineurium was carefully dissected from underlying fascicles using a refined microsurgical technique described in previous publications [15,16,17,18,19,20,21,22,23]. All prepared patches were evaluated for structural integrity; those with any signs of damage were excluded.

### 2.3. Human Amniotic Membrane

The placenta-derived amniotic membrane (hAM) (AmnioBand^®^ Viable, 2 cm × 4 cm; UPC: 840045714049 and lyophilized AmnioBand Membrane, 2 cm × 4 cm; UPC: 840045712908) was supplied by the Musculoskeletal Transplant Foundation (MTF, Edison, NJ, USA), our well-established partner. The hAM was shipped under sterile conditions on dry ice. Upon arrival, it was stored at −86 °C in a Glacier^®^ Ultralow Temperature Freezer (Thermo Fisher Scientific, Waltham, MA, USA) (Figure 1B).

### 2.4. Surgical Procedure

All surgical procedures were conducted under general anesthesia induced with 5% isoflurane (Terrell Isoflurane, Piramal Critical Care Inc., Bethlehem, PA, USA) and maintained at 1.5–3.0% via the Classic T3™ SurgiVet^®^ Vaporizer (Smiths Medical ASD Inc., St. Paul, MN, USA). Buprenorphine SR (1.2 mg/kg) was administered subcutaneously 15 min before incision to ensure analgesia. Rats were placed in lateral decubitus (left side down), and the right hind limb was prepared by shaving (Nair™, Church & Dwight Co., Ewing, NJ, USA). The skin was disinfected using 5% povidone–iodine (Betadine^®^, Purdue Pharma L.P., Stamford, CT, USA) and the animals were positioned on a circulating warm-water heating pad (T/Pump^®^, Gaymar Industries, Orchard Park, NY, USA). Using microsurgical instruments and an operating microscope (Wild M-691, Leica Microsystems, Wetzlar, Germany) at 20× magnification, a 3 cm skin incision was made in the gluteal region to expose the sciatic nerve between the gluteus superficialis and biceps femoris muscles. A crush injury was induced 1 cm proximal to the nerve bifurcation by applying a straight mosquito hemostat (Walter Lorenz^®^, Jacksonville, FL, USA) for 5 min to a 1.5 mm segment. The closing pressure of 17.4 N/mm, corresponding to the second notch of the instrument, was selected based on previous standardization experiments [24]. This pressure consistently produced a partial and reproducible injury consistent with axonotmesis, without causing complete transection. The pressure was measured using the MTS 858 Bionix^®^ Tabletop Test System (MTS Systems Corporation, Eden Prairie, MN, USA) and analyzed with MTS TestSuite™ Multipurpose Testing Software, version 4.0 (MTS Systems Corporation, Eden Prairie, MN, USA). Depending on group allocation, the injured nerve was either wrapped with hAM (Figure 2B), hEP (Figure 2C), or left without protection (control group). To prevent the risk of displacement of the hAM or hEP, the patches were fixed using two additional sutures (10-0 nylon suture, Ethicon Inc., Raritan, NJ, USA) to the fascia of the underlying gluteal muscle. The muscles were approximated using a 4-0 interrupted Vicryl suture (Ethicon Inc., Somerville, NJ, USA), while the skin was washed using a 5% Povidone–Iodine solution (Betadine^®^, Purdue Pharma L.P., Stamford, CT, USA), closed using interrupted 5-0 Monocryl sutures (Ethicon Inc., Raritan, NJ, USA). Postoperative care included antibiotic cream application (Neosporin^®^, Johnson & Johnson, New Brunswick, NJ, USA) and 24 h monitoring.

#### Postsurgical Supportive Treatment

During the initial 24 h period, each rat was isolated and equipped with a protective collar to prevent self-inflicted wounds. Following this period, the collar was removed, and the rat was returned to its original cage. Pain management involved administering Buprenorphine (0.1 mg/kg) twice daily for the first two postoperative days. Throughout the first 14 days after surgery, the rats were subjected to daily physical examinations to monitor the condition of the surgical site, signs of morbidity, changes in eating or drinking patterns, weight fluctuations, and inability to perform physical activities. Indicators of pain or distress, such as rough hair or hunched posture, were carefully observed. An experienced board-certified veterinarian from the College of Veterinary Medicine, University of Illinois at Chicago (USA), performed assessments to ensure animal well-being.

### 2.5. Experimental Groups and Study Design

Thirty-six athymic nude rats (*Crl:NIH-Foxn1^rnu^*) were randomly assigned to three experimental groups (*n* = 12/group). Each group was further subdivided for the 6-week (*n* = 6/subgroup) and 12-week (*n* = 6/subgroup) study durations (Table 1). In Group 1, the sciatic nerve was subjected to a crush injury, and no protective wrapping was applied (control). In Group 2, the sciatic nerve crush injury was followed by the hEP application. In Group 3, the crushed sciatic nerve segment was wrapped with the hAM. Assessments were conducted at 1, 3, and 6 weeks for the 6-week study and at weeks 1, 3, 6, 9, and 12 weeks for the 12-week study (Table 1).

### 2.6. Functional Tests and Histomorphometric Analysis

#### 2.6.1. Functional Motor Assessment by Toe-Spread Test

Motor functional recovery of the right sciatic nerve was evaluated using the standardized Toe-Spread test, as previously described [23,25]. Briefly, when a rat is suspended by the tail, it naturally extends and abducts the toes of the hindfoot. The assessment was conducted on a 0–3 scale. Each rat was evaluated three times per assessment point at the designated time intervals.

#### 2.6.2. Functional Sensory Assessment with Pinprick Test

Sensory recovery was evaluated using the Pinprick test, as previously described [23,25]. A noxious pinch was applied using Adson’s toothed forceps (Walter Lorenz^®^, Jacksonville, FL, USA). The response was assessed based on limb withdrawal and/or vocalization, with controlled pressure applied for a specific duration to ensure stimulation of the skin without affecting deeper tissues. Sensory function was graded on a 0–3 scale. Each rat underwent the test three times per evaluation point. Additionally, post-test monitoring was conducted to check for signs of ecchymosis, wounds, edema, or changes in skin color.

#### 2.6.3. Macroscopic Assessment of the hEP and hAM at the Sciatic Nerve Injury Site 

At the designated study endpoints of 6 and 12 weeks, animals were euthanized using SomnaSol™ (Henry Schein Inc., Melville, NY, USA). Post mortem, a 3 cm incision was made in the gluteal region of the right hind limb to harvest sciatic nerve samples from both the control (no protective wrapping) and the treatment groups (protected with either hEP or hAM). Additionally, straight incisions were made along the posterior aspect of both lower limbs to collect gastrocnemius muscle samples. All harvested tissues were processed for histopathological and immunofluorescence evaluation. The sciatic nerves were specifically examined for tissue adhesion, local inflammatory responses, and the condition of the hEP and hAM patches, including their structural integrity, morphology, and degree of vascularization.

#### 2.6.4. Assessment of Muscle Denervation Atrophy by Gastrocnemius Muscle Index 

Muscle denervation atrophy was assessed by the Gastrocnemius Muscle Index (GMI) at the endpoints of the 6- and 12-week study. The wet weight of the gastrocnemius muscle (GM) was measured using a digital analytical balance (Ohaus Explorer™, OHAUS, Parsippany, NJ, USA). The GMI was calculated by dividing the wet weight of the right GM (operated side) by the wet weight of the left GM (contralateral non-operated side). The denervation atrophy recovery of the GM on the operated side was presented as a percentage value, where a GMI of 100% corresponded to complete restoration of muscle weight.

#### 2.6.5. Histomorphometric Analysis

Samples of the proximal, crushed, and distal sciatic nerve sections from three study groups—no protection, hEP application, and hAM application—were collected. Following excision, the samples were immediately immersed in 2.5% glutaraldehyde for primary fixation. Subsequently, a 4% aqueous osmium tetroxide solution was employed for post-fixation, adhering to the manufacturer’s protocol. Toluidine Blue Solution (Thermo Fisher Scientific, Waltham, MA, USA) was utilized to stain 1 μm thick cross-sections for histological evaluation. The slides were examined under a light microscope, Leica DM 5500B Automated Upright Microscope (Leica Microsystems, Wetzlar, Germany), with a high objective lens (magnification ×100) equipped with a digital camera, Leica DFC290 HD Color Digital FireWire Camera (Leica Microsystems, Wetzlar, Germany). Quantitative morphometric analysis was performed using Image-Pro Plus, version 4.0 (Media Cybernetics, Rockville, MD, USA), selecting six random, non-overlapping regions per section. ImageJ, version 1.54j (National Institutes of Health, Bethesda, MD, USA) was used to assess key histomorphometric parameters, including myelin thickness (μm), axonal density (axons/μm^2^), fiber diameter (μm), and percentage of the myelinated nerve fibers (%).

### 2.7. Assessment of Immune Responses

Paraffin-embedded cross-sections of the sciatic nerve, including both the crush and distal segments, were subjected to immunofluorescent staining to assess the expression of neurogenic (GFAP, Laminin B, NGF, S-100), angiogenic (VEGF, vWF), and immunogenic (HLA-DR and HLA-I) markers. Nerve sections were mounted on glass slides and deparaffinized using sequential incubations in fresh xylene, followed by rehydration through a graded ethanol series and a final rinse in distilled water. Slides were then washed for 2 min in Tris-buffered saline containing 0.1% Tween-20 (TBS-T; Agilent Technologies, Santa Clara, CA, USA) on an orbital shaker. Fixation was carried out with cold acetone for 8 min, followed by three additional washes in TBS-T (5 min each) under gentle agitation. To prevent non-specific binding, slides were incubated with 10% goat serum for 1 h at 4 °C in a humidified chamber. Primary antibodies were then applied at an optimized dilution of 1:50 in antibody diluent, and slides were incubated overnight at 4 °C in the dark. The following primary antibodies were used: GFAP (anti-GFAP, ab68428, RRID: AB_1209224, Abcam, Cambridge, UK), Laminin B (Laminin, RRID: AB_2133633, Invitrogen, Waltham, MA, USA), NGF (Anti-NGF beta, RRID: AB_10856084, Bioss Antibodies, Woburn, MA, USA), S-100 (S100 4C4.9, RRID: AB_795376, Invitrogen, Waltham, MA, USA), VEGF (anti-VEGF VG1, RRID: AB_10001947, Novus Biologicals, Englewood, CO, USA), vWF (VWF, RRID: AB_10642840, Proteintech, Rosemont, IL, USA), MHC class I (HLA class I APC, RRID: AB_1557426, Proteintech, Rosemont, IL, USA), and MHC class II (BD Pharmingen^TM^ Purified HLA-DR, DP, DQ, RRID: AB_395939, BD Biosciences, Franklin Lakes, NJ, USA). Following primary antibody incubation, slides were rinsed three times with TBS (5 min each) on an orbital shaker. The secondary antibodies, IgG Alex Fluor^TM^ 488 (RRID: AB_2534069, Invitrogen, Waltham, MA, USA) and IgG Alexa Fluor^®^ 594, ab150132 (RRID: AB_2810222, Abcam, Cambridge, UK), were diluted by 1:500 in antibody diluent and applied for 1 h at 4 °C in the dark in a humidity chamber. After secondary incubation, slides were washed three times with PBS (5 min each), counterstained with DAPI, and mounted using Fluoromount-G^®^ (SouthernBiotech, Birmingham, AL, USA). Fluorescence was visualized using an upright confocal microscope (Leica TCS SP2, RRID: SCR_020231, Leica Microsystems, Wetzlar, Germany) equipped with a digital camera (QImaging^®^ Retiga-2000R CCD, QImaging, Surrey, BC, Canada). Image analysis was performed using Image-Pro Plus, version 4.0 (Media Cybernetics, Rockville, MD, USA). Immunoreactivity was semi-quantitatively scored based on fluorescence intensity: 0 = no signal; 1 = weak; 2 = moderate; 3 = strong.

### 2.8. Statistical Analysis

The GraphPad Prism, version 9.0.0 (GraphPad Software, San Diego, CA, USA) software was used for statistical analysis. To define statistical significance, one-way or two-way ANOVA tests (JMP Pro, version 17.0 (SAS Institute Inc., Cary, NC, USA)) followed by Tukey’s post hoc test were performed, allowing group comparisons. Statistical significance was considered at *p*-value < 0.05 and was marked with asterisks: * *p* < 0.05, ** *p* < 0.01, *** *p* < 0.001, **** *p* < 0.0001.

## 3. Results Following Sciatic Nerve Crush Injury in a 6-Week Study

### 3.1. Toe-Spread Test

**Figure 3:**
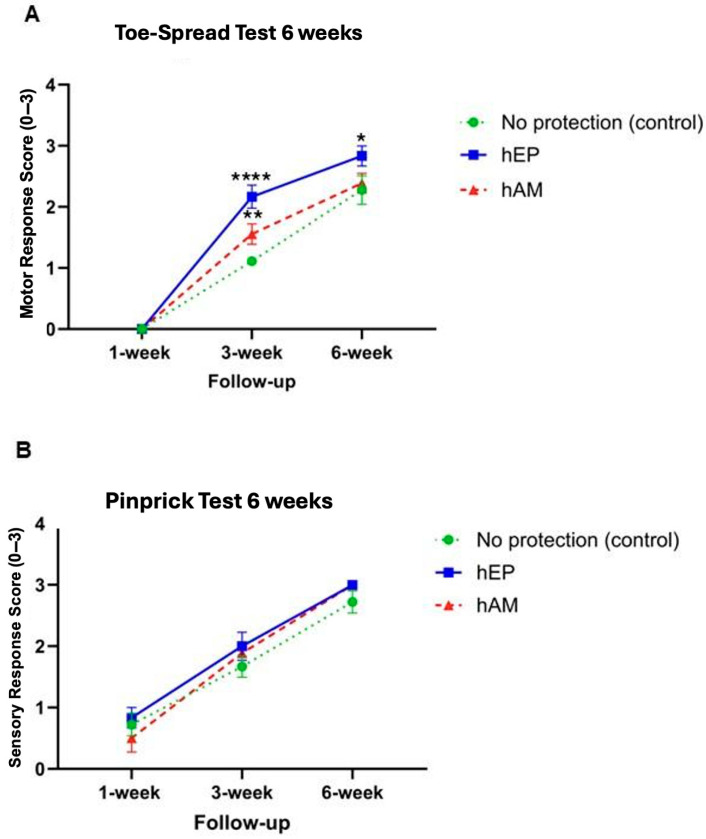
Functional sensory and motor assessment of nerve regeneration by Toe-Spread and Pinprick tests in the 6-week study at 1, 3, and 6 weeks following sciatic nerve crush injury. (**A**) Significantly better motor outcomes were observed when comparing the group that underwent hEP application with the no-protection group and the hAM wrapping group at the 3-week study point following nerve repair. Furthermore, statistically significant differences were evident between the no protective wrapping and hEP groups at the 6-week study endpoint. (**B**) This 6-week study did not yield statistically significant data from the Pinprick test. Both tests were scored using a 3-point scale (0–3), where higher values indicate better functional recovery: for the Pinprick test, 0 = no response, 1 = withdrawal response, 2 = withdrawal with some nocifensive behavior, and 3 = brisk and immediate nocifensive reaction; for the Toe-Spread test, 0 = no movement, 1 = weak or partial toe spreading, 2 = moderate spreading, and 3 = full, strong toe spreading. The graphs represent mean values with SEM; statistical significance is marked with asterisks: * *p* < 0.05, ** *p* < 0.01, **** *p* < 0.0001.

The Toe-Spread test was performed at designated intervals to evaluate motor recovery following sciatic nerve crush injury (Figure 3A). The application of hEP resulted in the best functional outcomes across all tested time points. Significantly, higher Toe-Spread scores were observed in the hEP group at the 3- and 6-week follow-ups. At the 3-week follow-up, motor function was markedly improved in the hEP group compared to the hAM patch group (*p* < 0.01) and also higher when compared to the control group without protective wrapping (*p* < 0.0001) (Figure 3A). At the 6-week follow-up, motor recovery remained significantly better in the hEP group compared to the control group (*p* < 0.05). No statistically significant difference was found between the hEP and hAM groups at this time point (Figure 3A).

### 3.2. Pinprick Test

The Pinprick test assessed sensory function recovery. The results did not differ significantly across the evaluated time points; however, the hEP-wrapped group tended toward higher Pinprick scores compared to the control group without protective wrapping and the hAM group, particularly at the 1- and 3-week follow-ups (Figure 3B).

### 3.3. Gastrocnemius Muscle Index

**Figure 4:**
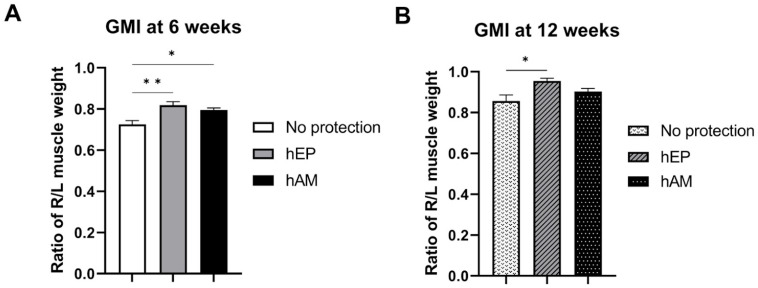
The assessments of muscle denervation atrophy by gastrocnemius muscle index (GMI) at 6 and 12 weeks following sciatic nerve crush injury. Significantly lower outcomes in the GMI were observed in the no protective wrapping group at both (**A**) the 6-week and (**B**) 12-week follow-up evaluations when compared to the hEP group. Data presented as mean ± SEM. A two-way ANOVA test for group comparison defined statistical significance, * *p* < 0.05, ** *p* < 0.01.

The GMI analysis demonstrated significantly higher values in the hEP group compared to the hAM group (*p* < 0.05), as well as compared to the control group (*p* < 0.01) (Figure 4A).

### 3.4. Myelin Thickness

At the proximal site, the myelin thickness was significantly greater in the hAM group compared to both the hEP and control groups (*p* < 0.0001), while no significant difference was observed between the hEP and control groups. At the crush site, the hAM group showed a significantly higher myelin thickness than both the hEP group (*p* < 0.0001) and the control group (*p* < 0.0001), and the hEP group exhibited significantly higher values than the control group (*p* < 0.05). At the distal site, the myelin thickness in the hEP group was significantly lower than in both the control group (*p* < 0.0001) and the hAM group (*p* < 0.001) (Table 2).

### 3.5. Fiber Diameter

The analysis of the proximal segment presented a higher average fiber diameter in the hEP group compared to the hAM wrap and control groups. However, these differences were not statistically significant. In the crush region, the hAM group demonstrated the largest fiber diameter, which was significantly greater than that observed in the hEP group (*p* < 0.0001). Furthermore, the hEP group exhibited a significantly larger fiber diameter compared to the control group (*p* < 0.05). The hAM group also showed a significant increase in the fiber diameter relative to the control (*p* < 0.0001). Conversely, in the distal segment, the hEP group presented a significantly larger fiber diameter than both the hAM group (*p* < 0.0001) and the control group (*p* < 0.0001) (Table 2).

### 3.6. Percentage of Myelinated Fibers

The proximal myelinated fiber percentage was significantly higher in both the hEP and hAM wrap groups compared to the group without protective wrapping (*p* < 0.001 and *p* < 0.01, respectively). At the crush site, a significant difference was observed between the hAM and hEP wrap groups (*p* < 0.05). Furthermore, the distal segment showed a significantly higher proportion of myelinated fibers in the hEP group compared to the hAM group (*p* < 0.01) (Table 2).

### 3.7. Axonal Density

At the proximal site, the axonal density was significantly higher in the hEP group compared to both the hAM group (*p* < 0.05) and the control group (*p* < 0.01). The hAM group also showed a significant increase compared to the control (*p* < 0.05). At the crush site, the hEP group exhibited a significantly greater axonal density than both the hAM group (*p* < 0.05) and the control group (*p* < 0.01). No significant differences in the axonal density were found between groups at the distal site (Table 2).

### 3.8. GFAP Expression

GFAP expression was highest in the hEP group compared to the hAM and control groups at the crush injury site and the distal nerve end. However, these differences were not statistically significant (Table 3).

### 3.9. Laminin B Expression

**Figure 5:**
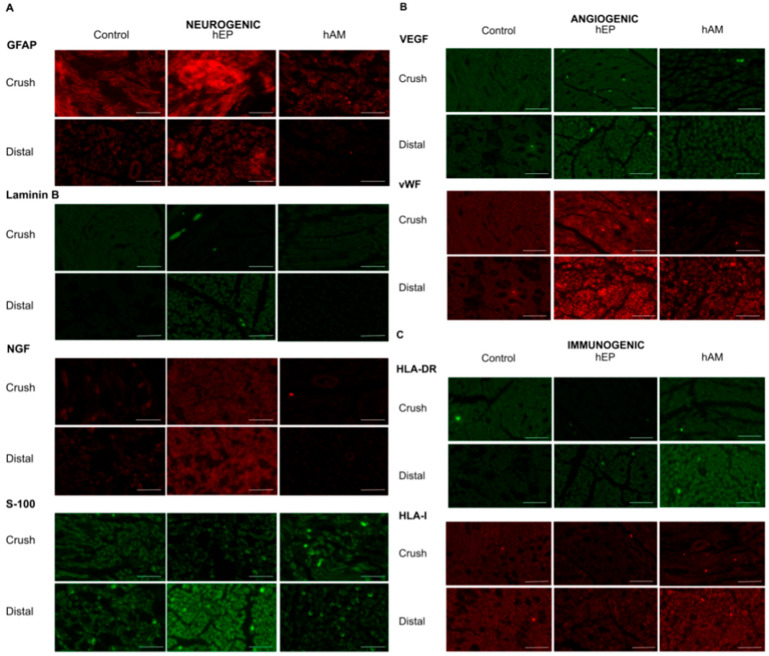
Immunofluorescence analysis of selected neurogenic (GFAP, Laminin B, NGF, S-100), angiogenic (VEGF, vWF), and immunogenic (HLA-DR, HLA-I) markers assessed at the crush and distal sites at 6 weeks following traumatic nerve injury. (**A**) GFAP expression was highest in the hEP group, indicating increased astroglial response. Laminin B expression was significantly elevated at the distal site in the hEP group compared to both the control and hAM groups, reflecting its role in nerve regeneration. The hEP group revealed the highest NGF expression, supporting its neurotrophic effect. S-100 expression was significantly increased in the distal section of the hEP group, suggesting enhanced Schwann cell-mediated nerve repair. (**B**) VEGF expression was highest in the hEP group. vWF expression was significantly elevated at the crush site in the hEP group compared to the control and hAM groups, supporting the angiogenic activity associated with hEP. (**C**) HLA-DR expression was highest at the crush site in the control group and lowest in the hEP group, suggesting a reduced immunogenic response. Similarly, HLA-I expression was lowest in the hEP group, further indicating its low immunogenic profile. Magnification 200×, scale bar 20 μm.

Laminin B expression was comparable at the crushed injury site among the control (no protective wrapping), hEP, and hAM groups (Table 3). However, at the distal nerve end, the expression of Laminin B was significantly higher in the hEP group compared to the hAM group (*p* < 0.05), and hEP reached higher values than the control group (Figure 5).

### 3.10. NGF Expression

NGF expression was highest in the hEP group compared to the hAM and control groups at the crush site and the distal nerve end. However, these differences were not statistically significant (Table 3, Figure 5).

### 3.11. S-100 Expression

S-100 expression at the crushed injury site was comparable between the groups treated with hEP and hAM; both showed higher values than the control group, although the differences were not statistically significant (Table 3). However, at the distal nerve end, S-100 expression was the highest in the hEP group, which was significantly better than in the hAM group (*p* < 0.05). The control group presented intermediate values (Figure 5).

### 3.12. VEGF Expression

VEGF expression at the crush injury site was highest in the hEP group compared to the hAM and control groups. A similar pattern was observed at the distal nerve end, where the hEP group exhibited the highest VEGF expression, followed by the hAM group, and the lowest expression was noted in the control group. However, these differences were not statistically significant (Table 3, Figure 5).

### 3.13. vWF Expression

At the 6-week follow-up, there was a significant increase in the expression of vWF observed at the crush injury site in the group treated with hEP compared to the group supported with hAM (*p* < 0.05) (Table 3). At the distal nerve end, the group treated with hEP showed the highest expression of vWF compared to the hAM and control groups (Figure 5).

### 3.14. HLA-DR Expression 

HLA-DR expression was lowest in the hEP group at both the crush injury site and the distal nerve end, followed by the hAM and control groups. However, no significant differences were observed between groups (Table 3, Figure 5).

### 3.15. HLA-I Expression 

At the 6-week follow-up, HLA-I expression at the crush repair site was lowest in the hEP group compared to both the control and hAM patch groups (Table 3). A similar trend was observed at the distal end, where the hEP group demonstrated the lowest HLA-I expression compared to the control and hAM groups. However, these differences were not statistically significant (Figure 5).

## 4. Results Following Sciatic Nerve Crush Injury in a 12-Week Study

### 4.1. Toe-Spread Test

**Figure 6:**
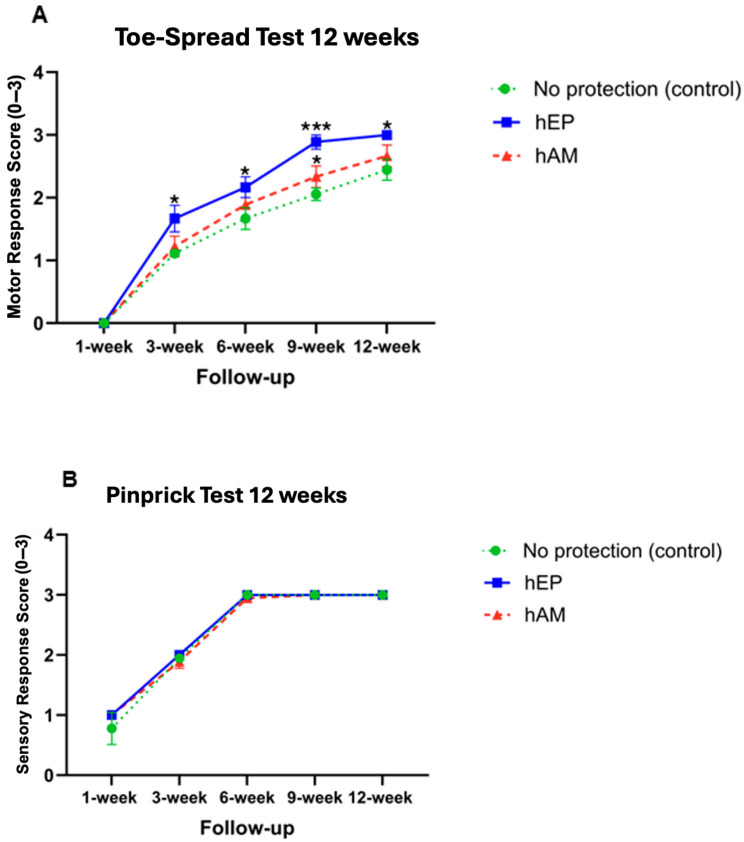
Functional evaluation of nerve regeneration by Toe-Spread and Pinprick tests in the 12-week study assessed over 1, 3, 6, 9 and 12 weeks following sciatic nerve crush injury. (**A**) The Toe-Spread test revealed a significant improvement in motor function in the group treated with hEP compared to the no-protection group at 3-, 6-, 9-, and 12-weeks post-injury. Notably, at the 9-week time point, the hEP group also demonstrated significantly better outcomes than the group treated with hAM. (**B**) In contrast, the Pinprick test did not show statistically significant differences between groups across the observation period. Both tests were scored using a 3-point scale (0–3), where higher values indicate better functional recovery: for the Pinprick test, 0 = no response, 1 = withdrawal response, 2 = withdrawal with some nocifensive behavior, and 3 = brisk and immediate nocifensive reaction; for the Toe-Spread test, 0 = no movement, 1 = weak or partial toe spreading, 2 = moderate spreading, and 3 = full, strong toe spreading. Data are presented as mean ± SEM. Statistical significance is indicated by asterisks: * *p* < 0.05, *** *p* < 0.001.

The hEP group consistently exhibited superior motor recovery compared to the control and hAM groups. At the 3-week follow-up, the hEP group showed significantly higher Toe-Spread scores than the control group (*p* < 0.05) (Figure 6A). This trend continued at the 6-week follow-up, where the hEP group outperformed the control group (*p* < 0.05). At the 9-week follow-up, significant differences in motor recovery were detected between the hEP group and both the control (*p* < 0.001) and the hAM group (*p* < 0.05). At the 12-week study endpoint, the hEP group demonstrated the best functional recovery with significantly higher Toe-Spread scores than the control group without protective wrapping (*p* < 0.05). No statistically significant difference was observed between the hEP and hAM groups at this final time point (Figure 6A).

### 4.2. Pinprick Test

The assessment of sensory recovery over the 12-week period revealed comparable outcomes across all groups at each follow-up (Figure 6B).

### 4.3. Gross Assessment 

**Figure 7:**
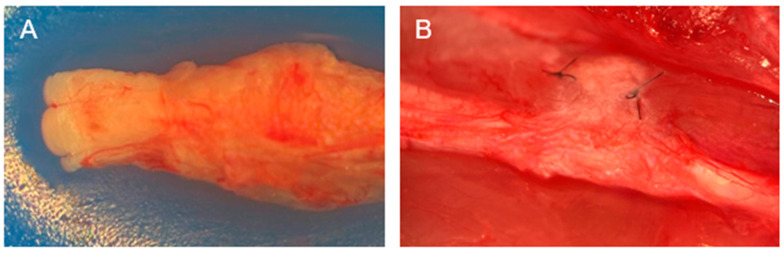
Macroscopic evaluation of the sciatic nerve crush site at the 12-week endpoint following hEP application. (**A**) Presence of fascicle-like structure. (**B**) Vascularization of the crushed part of the sciatic nerve.

The macroscopic evaluation at the 12-week endpoint revealed no signs of adhesion, fibrosis, or local inflammation. Fascicle-like structures were observed at the nerve crush injury site (Figure 7A). The hEP maintained structural integrity and good vascularization (Figure 7B). In contrast, the hAM was either minimally visible or completely undetectable.

### 4.4. Gastrocnemius Muscle Index

Statistically significant differences were confirmed between the control group without protective wrapping and the hEP wrap group (*p* < 0.05) (Figure 4B). Although higher GMI values were consistently observed in the hEP wrap group compared to the hAM wrap group, the differences did not reach statistical significance at week 12.

### 4.5. Myelin Thickness 

The myelin thickness at the proximal site was significantly lower in the hEP group compared to the hAM group (*p* < 0.0001), while the hAM group showed significantly higher values than the control group (*p* < 0.0001). At the crush site, a significantly lower myelin thickness was observed in the hEP group compared to both the hAM group (*p* < 0.05) and the control group (*p* < 0.0001), and the hAM group also exhibited significantly reduced values compared to the control group (*p* < 0.0001). At the distal site, the myelin thickness was significantly lower in the hEP group compared to the control group (*p* < 0.0001), but significantly higher than in the hAM group (*p* < 0.001) (Table 4).

### 4.6. Fiber Diameter

The outcomes of proximal fiber diameter presented a comparable trend to those observed in a 6-week study, demonstrating a significantly higher fiber diameter when comparing the hEP to the hAM wrap group (*p* < 0.0001) and the control (*p* < 0.0001). Conversely, at the crush site, fiber diameters were reduced in the hEP group relative to the hAM and control (*p* < 0.0001). Distally, the hEP also supported larger fiber diameters compared to the hAM (*p* < 0.001) (Table 4).

### 4.7. Myelinated Fibers Percentage

A significant increase in proximal myelinated fibers was observed in the hAM group compared to the hEP wrap group (*p* < 0.05). There was an increase in the proportion of myelinated fibers at the crush site following hEP application compared to the hAM wrap group; however, this difference was not statistically significant. Consistent with the 6-week study (Table 2), the percentage of distal myelinated fibers was significantly higher in the hEP wrap group compared to the hAM wrap group at the 12-week endpoint (*p* < 0.05) (Table 4).

### 4.8. Axonal Density

The proximal axonal density was significantly higher in the hEP group compared to both the hAM patch group (*p* < 0.01) and the control group (*p* < 0.0001). Although the crushed axonal density in the hEP group was higher than in the control group, this difference did not reach statistical significance. In contrast, the hAM group exhibited a significantly higher crushed axonal density compared to the control group (*p* < 0.05). In the distal nerve segment, the hEP group demonstrated the highest axonal density when compared to the hAM and control groups; however, these differences were not statistically significant (Table 4).

### 4.9. GFAP Expression

GFAP expression at the crush site was significantly higher in the hEP group compared to the hAM group (*p* < 0.05) and remained elevated compared to the control group (Table 5). However, within the distal nerve end, the expression of GFAP was highest in the control group, followed by the hEP group, and lowest in the hAM group, though these differences were not statistically significant.

### 4.10. Laminin B Expression

At the crush site, the expression of Laminin B was significantly higher in the control group compared to the hEP group (*p* < 0.05). However, the hEP group demonstrated significantly higher expression levels than the hAM group (*p* < 0.05) (Table 5). At the distal nerve end, the expression of Laminin B was highest following hEP application compared to both the control and hAM groups. However, these differences did not reach statistical significance.

### 4.11. NGF Expression

A significant increase in the expression level of NGF was observed at the crushed injury site when the group wrapped with hEP was compared to both the hAM group and the control group (*p* < 0.05) (Table 5). At the distal nerve end, a similar trend was observed, with the highest NGF expression in the hEP group, followed by the control group and the hAM group. However, the differences were not statistically significant.

### 4.12. S-100 Expression

Within the crushed site of the nerve injury, the highest S-100 expression was observed in the hEP group compared to both the hAM and control groups. No statistically significant differences were detected between groups (Table 5). At the distal nerve end, the hEP group exhibited significantly higher S-100 expression compared to the hAM group (*p* < 0.05), while the control group remained at an intermediate level.

### 4.13. VEGF Expression

VEGF expression at the crush injury site was highest in the hEP group, followed by the control group, and lowest in the hAM group. At the distal nerve end, VEGF expression was again highest in the hEP group, followed by the hAM group, and lowest in the control group. None of these differences were statistically significant (Table 5).

### 4.14. vWF Expression

vWF expression at the crush injury site remained higher in the hEP group compared to the hAM group, although the difference was not statistically significant. Similarly, the hEP group exhibited higher vWF expression than the unwrapped control group (Table 5). The same trend was observed at the distal nerve end, with the highest vWF expression in the hEP group, followed by the hAM group, and the lowest in the control group.

### 4.15. HLA-DR Expression

The expression of HLA-DR at the crush site was significantly lower in the hEP group compared to the hAM group. Moreover, the hEP group demonstrated markedly reduced HLA-DR expression relative to the control group (*p* < 0.05) (Table 5). At the distal nerve end, HLA-DR expression was significantly lower in the hEP group compared to the hAM group (*p* < 0.05), and the hEP group exhibited lower expression than the control group.

### 4.16. HLA-I Expression 

The hEP group exhibited significantly lower HLA-I expression at the crush injury site compared to the control group (*p* < 0.05). HLA-I expression in the hEP group was comparable to the hAM group (Table 5). No significant differences were observed in the expression of HLA-I between the experimental groups at the distal end, and the values were comparable.

## 5. Discussion

Acute nerve crush injuries occur as a result of traffic accidents, fractures, natural disasters, and combat injuries [24,26]. Furthermore, iatrogenic nerve injuries due to surgical procedures contribute to approximately 17% of nerve injuries [27]. Despite improvements in surgical techniques and research, the reported outcomes rarely show the full recovery of sensory and motor function. Therefore, the exploration of novel approaches to improve nerve regeneration is needed [28,29].

Following a crush injury, the nerve is subjected to an inflammatory response followed by Wallerian degeneration, demyelination, and nerve regeneration [30], where the Schwann cells play a critical role in modulating the immune response and promoting axonal regeneration through neurotrophic support [31]. Several factors influence peripheral nerve repair outcomes, including the injury type, location, extent, timing of surgical intervention, repair technique, fascicle alignment, and patient-specific comorbidities [32].

Moreover, depending on the severity of the nerve trauma, the crushed nerves may be subjected to the development of inflammation, fibrosis, and adhesions with the surrounding tissues. Therefore, different biological and synthetic materials were introduced to facilitate regeneration and protect nerves from the surrounding tissues [33,34]. However, the assessment of the efficacy of different biomaterials remains challenging, as nerve transection injuries cause considerably more trauma compared to nerve crush injuries [35] where the axonal structure remains intact, allowing for the fascicle regrowth along the pre-existing pathways. It is important to emphasize that the crush injury used in this study represents axonotmesis, a type of nerve injury in which the axonal continuity is disrupted but the connective tissue sheaths remain preserved. This mechanism enables earlier and more organized functional recovery compared to neurotmesis, or complete nerve transection [36]. Given these advantages, the nerve crush injury model was selected for this study, as it allows one to assess nerve regeneration with minimal disruption to the surrounding tissue. Various synthetic materials or naturally derived products were used to prevent perineurial scarring and improve the regeneration of the injured nerves, such as vein grafts, fat or muscle flaps, collagen or chitin tubes, silicon sheets, and the human amniotic membrane. However, many of these materials are associated with limited availability, immunogenicity, and biodegradability, which may compromise regeneration outcomes [37,38].

Over the past decade we have investigated the application of the epineural sheath (ES) in peripheral nerve repair in the rat, rabbit, and sheep models [5,17,18,20,39]. The epineural sheath (ES) is a naturally occurring tissue of a neural origin, has low immunogenicity, and provides a favorable microenvironment for nerve regeneration due to its high expression of neurogenic and angiogenic factors [5]. Our previous studies testing the application of epineural conduits or tubes following nerve injuries revealed encouraging results in enhancing peripheral nerve regeneration [16,18,19,40]. The standardized methodology used in this study, grounded in more than 20 years of expertise in experimental nerve regeneration, ensures consistent comparisons and highlights the translational potential of hEP as a therapeutic strategy. This study aimed to evaluate the neuroprotective effects of hEP, a human epineurium-derived material, in a rat model of peripheral nerve crush injury and compare its efficacy to hAM. The hEP is designed to foster an optimal environment for nerve regeneration and healing. It serves as a protective barrier against adhesions, reduces inflammation, prevents excessive fibrosis, and promotes vascularization. The epineural sheath, as an allogenic patch lacking Schwann cells, provides an ideal immunological material that does not require immunosuppression and enhances nerve regeneration [5,17,18,19,20].

Among commercially available biological nerve-protecting materials, the human amniotic membrane (hAM) is proposed and is primarily used for the enhancement of wound healing. It acts as a physical barrier, restricting fibrous tissue invasion, thus protecting regenerating tissue from scarring [13,41]. Both the hAM and hEP are characterized by low immunogenicity due to their low expression of MHC and HLA antigens. Moreover, the hAM has been proven to provide anti-inflammatory, antiadhesive, proangiogenic, and antimicrobial properties through the secretion of various active growth factors and cytokines [42]. The hAM has been shown to be safe and effective in clinical applications, mainly in ophthalmology and dermatology, and its biological properties have been exploited in guided bone regeneration [14,41]. Although recent studies demonstrate the beneficial effects of the hAM on peripheral nerve regeneration, the specific mechanism of action of the amniotic membrane in axon regeneration is still not fully understood [8,43]. Further research is needed to better understand its role in peripheral nerve injury management.

In this study, the hEP was selected due to its natural origin and low immunogenic profile. The macroscopic evaluation at the study endpoints of both 6 and 12 weeks revealed the intact structure of a hEP and, more importantly, a network of blood vessels was present on its surface. On the contrary, the hAM was totally absorbed into the surrounding tissue. Remarkably fewer adhesions were observed for the hEP and hAM groups compared to the control group, which is consistent with previous research [12,18].

The gross assessment conducted at a 12-week endpoint demonstrated the structural integrity and persistence of the hEP at the implantation site. Notably, the surface of the hEP exhibited visible neovascularization, indicating active angiogenesis and integration with the host tissue, whereas the hAM was largely resorbed into the surrounding tissue, with minimal structural remnants observed. Both the hEP and hAM groups exhibited a markedly reduced incidence of perineural adhesions and neuroma formation compared to the unprotected control group, findings consistent with previous reports [12,25].

Our study evaluated both motor and sensory functional recovery using standard Toe-Spread and Pinprick tests over 6- and 12-week periods following sciatic nerve crush injury. Interestingly, the Toe-Spread test revealed the most pronounced improvements in the group treated with a hEP, consistently across both observation periods. These results suggest that the hEP effectively supports the restoration of motor function, with noticeable benefits apparent as early as three weeks post-injury and sustained over a 12 weeks observation time. The superior motor outcomes observed in the hEP group highlight its potential as a supportive nerve protector after nerve injury repair.

In contrast, sensory recovery assessed by the Pinprick test did not show significant differences between groups at any time point, although a trend toward faster recovery was noted in the hEP group during the initial phase of recovery. At the final 12-week follow-up, sensory recovery in the hEP and hAM groups was comparable, indicating that while both biological membranes may support long-term regeneration, the hEP application may offer an advantage in accelerating early sensory recovery. The observed differences between motor and sensory functional recovery may be attributed to the intrinsic properties of motor and sensory neurons and their respective regenerative capacities following injury. Sensory neurons exhibit a higher regenerative potential and appear to be less responsive to biological scaffolds. Moreover, sensory fibers are known to regenerate more rapidly than motor fibers, which could explain the absence of statistically significant differences in sensory recovery between groups [44].

Muscle denervation atrophy remains a major challenge following nerve injury, as prolonged disuse can lead to irreversible muscular degeneration. The increased GMI values in the hEP-treated group indicate that this approach may help mitigate muscle atrophy, preserving muscle architecture and function during the critical healing period. This is particularly important for successful reinnervation and functional recovery, as muscles that maintain structural integrity may facilitate the regain of the pre-injury function. These findings align with our previous research [25].

Parameters such as the fiber diameter, myelin thickness, axonal density, and the percentage of myelinated fibers are considered among the most reliable indicators of nerve regeneration. Light microscopy data revealed that the hEP consistently supported nerve regeneration, particularly by enhancing the axonal density and fiber diameter at both 6 and 12 weeks. At 6 weeks, the hEP significantly improved the axonal density at the proximal and crush sites, indicating early neurotrophic activity, later aligning with elevated GFAP and NGF expression. Importantly, it also showed the highest percentage of myelinated fibers proximally and distally, suggesting not only axonal outgrowth, but also progress in remyelination. At the crush site, the percentage of myelinated fibers in the hEP group was significantly lower than in the hAM group. However, at 12 weeks, this parameter was the highest in the hEP group, suggesting its pro-regenerative properties, though the difference was not statistically significant. While the myelin thickness was greater in the hAM group, hEP-treated nerves exhibited larger fiber diameters and a higher proportion of myelinated axons, especially at the distal to crush injury sites. This discrepancy may reflect a more gradual and physiological pattern of the remyelination process, as supported by increased S-100 and NGF expression. At 12 weeks, the hEP maintained its regenerative advantage, with a superior axonal density proximally and significantly larger fiber diameters at the proximal and distal site. The myelin thickness in the hEP group showed a different trend at the distal site, with significantly greater values than those observed in the hAM group. These findings suggest that the hEP promotes sustained, structured nerve repair, even if myelin maturation appears slower [23,45]. Considering the regional differences in the myelin thickness in the injured axon, all study groups exhibited a greater myelin thickness at the proximal site compared to the distal segments. This effect might be associated with swelling due to enhanced axonal transport, swollen mitochondria, and the vacuolization of the axoplasm [46,47].

A detailed analysis of several key markers associated with neurogenesis, angiogenesis, and immunomodulation showed promising neuroregenerative effects of the hEP. Notably, the hEP wrap demonstrated the enhanced expression of GFAP, Laminin B, NGF, S-100, and VEGF at the crush injury site, particularly at the 12-week follow-up, suggesting improved neuronal support, axonal regeneration, and tissue repair. GFAP, a glial marker, is indicative of astrocyte activation and neuroinflammation, which plays a critical role in the regenerative response following nerve injury. Laminin B, a key component of the extracellular matrix essential for nerve regeneration, showed an interesting dual pattern. While its early expression was enhanced distally by hEP application, by 12 weeks, the control group exhibited the highest levels at the injury site. It remains unclear whether the delayed upregulation of Laminin B in the control group represents an alternative regenerative pathway triggered by the absence of external support or a compensatory mechanism activated at later stages of repair. The significant increase in NGF expression, a neurotrophic factor, further suggests that the hEP wrap fosters neuronal survival and axonal outgrowth. Moreover, elevated S-100 expression suggests enhanced Schwann cell activity, essential for myelination and nerve repair. Notably, angiogenic factors such as VEGF and vWF followed a consistent pattern across the study time points, with hEP-treated nerves maintaining the highest expression levels. This supports a pro-angiogenic role of the hEP wrap, potentially facilitating oxygen and nutrient delivery to regenerating axons, which is necessary for nerve regeneration while preventing complications such as tissue ischemia. One of the most compelling findings was the confirmation of a non-immunogenic profile of hEP, as evidenced by negligible HLA-DR and HLA-I expression throughout this study. This suggests that the hEP can be safely applied without triggering an adverse immune response, a crucial consideration for potential clinical applications. Compared to the hAM, the hEP emerges as a superior alternative, offering enhanced regenerative benefits while maintaining immune compatibility [18].

It should be acknowledged that this study has certain limitations. First, we employed only an acute nerve injury model, which may not fully replicate the pathological complexity of the chronic or segmental nerve damage observed in clinical scenarios. However, it is important to note that we included both short-term (6 weeks) and long-term (12 weeks) outcomes, providing a broader temporal perspective on regenerative changes. According to established rat-to-human age conversion scales, a 12-week period in rats approximates 8 years in humans [48], underscoring the translational relevance of our long-term observations. Future studies should consider chronic injury models or the use of larger animal models to better approximate human nerve repair conditions.

Considering its natural origin, ease of availability, low immunogenicity, and favorable biological profile, the human epineural patch (hEP) represents a valuable alternative to the human amniotic membrane in peripheral nerve repair. Its ability to reduce fibrosis, promote vascularization, and support both motor and sensory recovery underscores its translational potential. Our study successfully demonstrated the application of a hEP in repairing peripheral nerve damage, as assessed in an experimental sciatic nerve crush injury model, with positive outcomes observed up to 12 weeks post-repair. The results of our experimental study are promising and suggest that hEP can be particularly beneficial in primary nerve repair and in reinforcing tension-free anastomoses [25]. While further research exploring hEP efficacy in chronic injury models is needed, the current study confirmed that the hEP has the potential to serve as an ‘off-the-shelf’ solution, enabling rapid clinical use in peripheral nerve injury management.

## 6. Conclusions

This study demonstrated the regenerative potential of a hEP at 6 and 12 weeks after application to the sciatic nerve crush injury, tested in the experimental nude rat model. Our findings highlight the hEP’s ability to enhance nerve regeneration, promote functional recovery, and create a favorable microenvironment for axonal repair. Compared to the hAM, the hEP exhibited greater structural stability, superior neurogenic marker expression, and improved nerve morphometry, suggesting long-term nerve regeneration. The unique concept of the hEP introduces a novel ‘off-the-shelf’ product for nerve protection and the enhancement of nerve regeneration after trauma.

## Figures and Tables

**Table 1:** Outline of the experimental groups. In both the 6-week and 12-week studies, nerve regeneration evaluation following sciatic nerve crush injury was conducted across three conditions: control (no protective wrapping) (Group 1), hEP application (Group 2), and hAM wrapping (Group 3).

Experimental Group Number	Repair Method	Number of Athymic Nude Rats per Group
	6 weeks study	
1	no protective wrapping	*n* = 6
2	hEP	*n* = 6
3	hAM	*n* = 6
	12 weeks study	
1	no protective wrapping	*n* = 6
2	hEP	*n* = 6
3	hAM	*n* = 6

**Table 2:** Histological assessment of the proximal, crushed, and distal segments of the sciatic nerve at 6 weeks after crush injury.

6 Weeks Study				
Parameter	Location	Control (Mean ± SEM)	hEP (Mean ± SEM)	hAM (Mean ± SEM)
Myelin Thickness				
(μm)	Proximal	0.532 ± 0.016	0.571 ± 0.017	0.677 ± 0.018 ****
	Crush	0.372 ± 0.009	0.326 ± 0.008	0.460 ± 0.018 ****
	Distal	0.334 ± 0.007 ****	0.278 ± 0.008	0.320 ± 0.007 ****
% of Myelinated Fibers				
(%)	Proximal	84.711 ± 1.570	91.185 ± 0.628 ***	89.454 ± 0.865 **
	Crush	86.509 ± 0.621	84.102 ± 0.666	86.991 ± 0.864 *
	Distal	84.637 ± 0.852	86.191 ± 0.588 **	82.970 ± 0.719
Fiber Diameter				
(μm)	Proximal	11.770 ± 0.359	12.568 ± 0.213	12.006 ± 0.179
	Crush	8.029 ± 0.077 *	8.577 ± 0.104 ****	9.720 ± 0.190 ****
	Distal	8.196 ± 0.087	8.871 ± 0.117 ****	8.246 ± 0.096 ****
Axonal Density				
(axons/μm^2^)	Proximal	28.722 ± 3.477	41.111 ± 2.021 **	39.000 ± 1.690 *
	Crush	47.167 ± 2.306	60.556 ± 4.027 **	47.611 ± 2.432
	Distal	44.389 ± 2.171	47.444 ± 2.173	48.222 ± 1.821

Data presented as mean ± SEM. A two-way ANOVA test for group comparison defined statistical significance, * *p* < 0.05, ** *p* < 0.01, *** *p* < 0.001, **** *p* < 0.0001.

**Table 3:** Expression evaluation of selected neurogenic, angiogenic, and immunogenic markers assessed at the crush injury sites and distal ends by immunofluorescence staining at 6 weeks following nerve crush injury.

6-Week Study			
	Control	hEP	hAM
NEUROGENIC			
GFAP			
Crush	2.333 ± 0.333	2.833 ± 0.167	1.583 ± 0.083
Distal	1.500 ± 0.500	1.833 ± 0.601	0.583 ± 0.083
Laminin B			
Crush	1.167 ± 0.167	1.167 ± 0.333	1.167 ± 0.167
Distal	0.500 ± 0.000	1.500 ± 0.500 *	0.333 ± 0.167
NGF			
Crush	0.500 ± 0.289	1.250 ± 0.629	0.667 ± 0.167
Distal	0.333 ± 0.167	1.833 ± 0.601	0.500 ± 0.289
S-100			
Crush	1.667 ± 0.167	1.833 ± 0.333	1.833 ± 0.333
Distal	2.167 ± 0.167	2.500 ± 0.289 *	1.167 ± 0.441
ANGIOGENIC			
VEGF			
Crush	1.167 ± 0.441	1.833 ± 0.167	1.167 ± 0.441
Distal	1.167 ± 0.167	1.833 ± 0.333	1.333 ± 0.167
wVF			
Crush	1.167 ± 0.667	2.167 ± 0.167 *	0.583 ± 0.083
Distal	1.167 ± 0.167	2.500 ± 0.500	1.833 ± 0.167
IMMUNOGENIC			
HLA-DR			
Crush	1.500 ± 0.289	0.833 ± 0.601	1.333 ± 0.441
Distal	1.000 ± 0.289	0.833 ± 0.167	1.500 ± 0.500
HLA-I			
Crush	1.400 ± 0.430	0.900 ± 0.187	1.000 ± 0.274
Distal	1.700 ± 0.255	0.900 ± 0.292	1.500 ± 0.224

A two-way ANOVA test for group comparison was used to define statistical significance, * *p* < 0.05.

**Table 4:** Histological assessment of the proximal, crushed, and distal parts of the injured segment at 12 weeks after crushed nerve injury.

12 Weeks Study				
Parameter	Location	Control (Mean ± SEM)	hEP (Mean ± SEM)	hAM (Mean ± SEM)
Myelin Thickness				
(μm)	Proximal	0.664 ± 0.030	0.641 ± 0.020	0.881 ± 0.037 ****
	Crush	0.502 ± 0.023 ****	0.294 ± 0.008	0.351 ± 0.010 ****
	Distal	0.421 ± 0.026 ****	0.372 ± 0.012 ***	0.274 ± 0.007
% of Myelinated Fibers				
(%)	Proximal	89.582 ± 0.748	88.475 ± 1.607	92.128 ± 0.474 *
	Crush	88.713 ± 0.656	89.784 ± 0.548	87.881 ± 0.664
	Distal	88.479 ± 0.507	89.537 ± 0.431 *	87.262 ± 0.665
Fiber Diameter				
(μm)	Proximal	11.701 ± 0.166	14.142 ± 0.189 ****	11.859 ± 0.176
	Crush	10.322 ± 0.151 ****	7.677 ± 0.088	8.748 ± 0.086 ****
	Distal	8.409 ± 0.114	8.957 ± 0.093 ***	8.574 ± 0.093 *
Axonal Density				
(axons/μm^2^)	Proximal	27.778 ± 2.535	40.222 ± 1.832 ****	38.167 ± 1.172 **
	Crush	45.944 ± 3.845	56.056 ± 3.785	59.222 ± 2.697 *
	Distal	54.667 ± 2.974	59.333 ± 3.734	57.556 ± 3.781

Data presented as mean ± SEM. A two-way ANOVA test for group comparison defined statistical significance, * *p* < 0.05, ** *p* < 0.01, *** *p* < 0.001, **** *p* < 0.0001.

**Table 5:** Expression evaluation of selected neurogenic, angiogenic, and immunogenic markers assessed at the crush injury sites and distal ends by immunofluorescence staining at 12-week following nerve crush injury.

12-Week Study			
	Control	hEP	hAM
NEUROGENIC			
GFAP			
Crush	1.833 ± 0.601 *	2.500 ± 0.289	0.750 ± 0.433
Distal	1.667 ± 0.601	1.333 ± 0.167	0.333 ± 0.167
Laminin B			
Crush	2.333 ± 0.333	2.000 ± 0.289 *	0.583 ± 0.220 **
Distal	1.167 ± 0.441	1.667 ± 0.333	0.750 ± 0.382
NGF			
Crush	0.500 ± 0.289	1.750 ± 0.144 *	0.667 ± 0.333
Distal	0.667 ± 0.167	0.917 ± 0.300	0.167 ± 0.167
S-100			
Crush	1.667 ± 0.441	2.167 ± 0.167	1.667 ± 0.167
Distal	1.333 ± 0.333	2.000 ± 0.289 *	0.833 ± 0.167
ANGIOGENIC			
VEGF			
Crush	2.333 ± 0.667	2.667 ± 0.167	2.000 ± 0.500
Distal	1.500 ± 0.500	2.167 ± 0.601	1.833 ± 0.167
wVF			
Crush	1.667 ± 0.601	2.167 ± 0.833	2.000 ± 0.500
Distal	0.833 ± 0.333	1.500 ± 0.289	0.917 ± 0.546
IMMUNOGENIC			
HLA-DR			
Crush	2.500 ± 0.289 *	1.083 0.363	2.000 ± 0.577
Distal	1.333 ± 0.333	0.833 ± 0.167	2.333 ± 0.333 *
HLA-I			
Crush	2.000 ± 0.289 *	0.917 ± 0.300 *	2.333 ± 0.333 *
Distal	0.917 ± 0.363	0.833 ± 0.167	0.667 ± 0.220

A two-way ANOVA test for group comparison was used to define statistical significance, * *p* < 0.05, ** *p* < 0.01.

## Data Availability

All data generated or analyzed during this study are either included in this published article or are available from the corresponding author upon reasonable request.

## Abbreviations

The following abbreviations are used in this manuscript:

AAALAC	American Association for the Accreditation of Laboratory Animal Care
ACC	Animal Care Committee
CTR	Crush/Transection/Repair
GFAP	Glial Fibrillary Acidic Protein
GMI	Gastrocnemius Muscle Index
hAM	human Amniotic Membrane
hEP	human Epineural Patch
HLA-DR	Human Leukocyte Antigen—DR
HLA-I	Human Leukocyte Antigen—Class I
MTF	Musculoskeletal Transplant Foundation
NGF	Nerve Growth Factor
S-100	S-100 Protein
SEM	Standard Error of the Mean
VEGF	Vascular Endothelial Growth Factor
vWF	von Willebrand Factor
